# Enhancement of Dermal Delivery of Finasteride Using Microemulsion Systems

**DOI:** 10.15171/apb.2019.067

**Published:** 2019-10-24

**Authors:** Saeed Mohammad Soleymani, Anayatollah Salimi

**Affiliations:** ^1^Student Research Committee, Ahvaz Jundishapur University of Medical Sciences, Ahvaz, Iran.; ^2^Department of Pharmaceutics, Faculty of pharmacy, Ahvaz Jundishapur University of Medical Sciences, Ahvaz, Iran.; ^3^Nanotechnology Research Center, Ahvaz Jundishapur University of Medical Sciences, Ahvaz, Iran.

**Keywords:** Dermal drug delivery, Finasteride, Microemulsion, Permeability, Release

## Abstract

***Purpose:*** Finasteride is a pharmaceutical agent that treats hair loss and acne with hormonal patterns. Due to its poor water solubility, and the smaller surface area in comparison to total skin surface area, penetration of the drug into hair follicles and skin is low. The aim of this research was to formulate, characterize and evaluate in vitro skin permeability of finasteride microemulsions (MEs).

***Methods:*** Finasteride MEs were prepared using a pseudo-ternary phase diagram method with an appropriate ratio of oil mixture, surfactant-co-surfactant mixture and water. MEs containing 1% finasteride were prepared with a suitable amount of oily phase and surfactant and cosurfactant. The physicochemical properties of these MEs and in vitro skin permeability of MEs were evaluated.

***Results:*** The results showed that the mean droplet size range of ME samples was 5–17 nm and pH was 5.1–5.7. The viscosity of MEs ranged from 86.4–209.6 cps. The drug release profile showed that 49.510% of the drug was released (ME-F-6) over the 24 hours of the experiment. The kinetics of drug release from all selected MEs were approximately described by Higuchi and first-order modeling. All ME formulations with different compositions and properties significantly increased flux and permeability coefficient from rat skin. The selected MEs exhibit 99.9% finasteride after six months of storage.

***Conclusion:*** This study showed that any change in the content and composition of MEs could change the physical and chemical properties in addition to ME permeability parameters. The MEs increased permeability of the skin to finasteride.

## Introduction

Hair, in addition to the role of cuticle, is important cosmetically for human beings. Hair loss sometimes causes mental illness. Androgenic hair loss is one of the most common types of hair loss, which in men and women, is genetically predisposed, causing hair loss with a specific pattern.^1^ Androgenic hair loss, also called androgenetic alopecia, occurs because of the sensitivity of the field of follicles to androgens. Androgenic hair loss is the most common reason for hair loss in 70% of men and 40% of women. Head hair in humans, although having non-androgen-dependent growth, has a specific receptor for sex hormones, and therefore these hormones can affect hair loss. In men, the frontotemporal regions, or the front and back regions as well as the center of the head, feature such hormonal receptors, but in women, all hair of the head area has androgenic receptors. As a result, the pattern of hair loss in men begins from the front of the head (nodes) and then reaches the central area, while in women, there is usually hair thinning and spreading without hair strain. The typical pattern of male hair loss is because of genetic sensitivity to the effects of dihydrotestosterone (DHT) in certain areas of the scalp. Testosterone is converted to its active form, DHT, which induces hair loss and is linked to androgenic receptors in hair follicles, which results in compromised growth phase of the hair cycle. It is believed that DHT shortens the growth stage, or anagen, from a typical period of three to six years to several weeks or months. This causes the hair follicles to shrink gradually, reducing hair production and making hair smoother. DHT is produced by an enzyme called 5-alpha-reductase.^2^ Finasteride is a 5-alpha-reductase (II) inhibitor which is administered in the treatment of such hair loss and acne by pills that are either 1 or 5 mg in strength. The use of this drug systemically has side effects such as complications like gynecomastia, behavioral disorders and loss of libido. The drug has a molecular weight of 372.5 Da (Log p = 3). Its low solubility in water (11.7 mg/L) is another physicochemical feature of finasteride.^3^ Low molecular weight, short half-life (six hours) and ideal physicochemical properties means finasteride is suitable for use on the skin.

A very low dissolution of a drug may limit its efficacy. Therefore, increasing the solubility of the drug is effective for improving therapeutic efficacy and reducing the total dose of the drug, thereby minimizing its adverse side effects. Increasing solubility with surfactants is one of the most important techniques evaluated in this context. Research has shown that the most critical reason for increasing the dissolution of microemulsions (MEs) is the presence of “solubilizing sites” in the hydrophilic and lipophilic areas of superficial interphase films, which will enhance the dissolution of hydrophilic and lipophilic compounds. Additionally, the unique structure of phases in MEs contributes to the presence of more “dissolving regions”, which increases the loading capacity of the drug in MEs compared with solutions containing the same proportion of the same components.^4^ A semi-solid form of MEs is one of the new formulas used as a topical delivery system.^5,6^ MEs have many features unique as topical and transdermal drug delivery systems. First, the main advantage of these colloidal systems is that a large amount of the drug can be combined owing to the increased solubilization capacity, and therefore the thermodynamic activity in the skin increases with the gradient having a high concentration of the drug present in the ME for the skin. Second, the degree of drug penetration using ME carriers is improved based on the interaction of various components to increase the delivery of medication across the skin. Third, the main components, such as the water phase, oil phase and surfactant-co-surfactant mixtures, can be combined to increase the charge of the drug.^7^ Furthermore, it is believed that surfactants and oils interact within a ME via a bilayer lipid structure and act as permeation enhancers.^8^

Knowing the internal microstructures of ME systems, such as phase change behavior and drug release, are vital to solution capabilities. On the other hand, this feature also affects the production and fabrication of polymer nanoparticles from these systems.^9^

In this study, the design and creation of a suitable formulation of a ME was carried out. This ME designed with proper loading capacity, suitable release rate and acceptable stability, would enhance the proper passage of a drug through the skin. If MEs are able to raise the follicular absorption of a compound, therapeutic efficacy of the drug will definitely improve, as well.

The ultimate aim of this research was to formulate, characterize and evaluate *in vitro* skin permeability of a finasteride ME.

## Materials and Methods

### Materials

Finasteride was purchased from Soha Helal (Tehran, Iran). Propylene glycol, Span 20, Tween 80, oleic acid, sodium dihydrogen phosphate and sodium hydrogen phosphate were obtained from Merck (Darmstadt, Germany). Transcutol P was donated by the Gattefosse (Lyon, France). The dialysis bags used in the present study was purchased from Tuba Azma Co. (Tehran, Iran). All chemicals and solvents were analytical grade. In addition, fresh double distilled water was used in the experiments.

### Animals

Male Wistar rats weighing 150-170 g that were 10-12 weeks old were used in the present study, which was performed with the approval of the Animal Ethical Committee, Ahvaz Jundishapur University of Medical Sciences. Rat abdominal hair was removed without damage to the skin. Rat with high concentrations of thiopental were anesthetized and then sacrificed. Subcutaneous fat was cleansed at the inner surface of the skin with pure cold acetone. The thickness of the skin was measured with digital micrometers.

### Finasteride assay

Determination of the amount of Finasteride was carried out by an ultraviolet (UV) spectroscopy method at λmax= 224 nm in buffer phosphate (pH = 7) and methanol 2:1.

### Screening of oils, surfactants and co-surfactants for microemulsions

Transcutol P and oleic acid as oils, Tween 80, Span 20 as surfactants and Propylene glycol were selected as co-surfactant and Finasteride solubility with these agents was determined. As such, 5 mL of each of the ingredients was poured into a beaker and added to each finasteride solution at 25°C for 48 hours with stirring.^10^ The resulting mixture was centrifuged at a rate of 3000 rpm for 15 minutes and the solution and sediment were separated. The amount of the dissolved drug was determined at 224 nm wavelength by UV spectrophotometry.^11^

### Pseudo-ternary phase diagram construction

The pseudo-ternary phase diagrams were created based on the information obtained from previous studies using Span 20 and Tween 80 surfactants, propylene glycol co-surfactant and the oil phase containing pleic acid and Transcutol P in ratios of 3:1 and 2:1, respectively. Based on the factorial design and pre-formulation trials with three variables at two levels for each variable, eight formulations were selected as presented in Table 1. Two levels of up-and-down were considered for each variable, and the variables in this study were the ratio of surfactant to co-surfactant (3:1 and 1:2), oil proportion (10 and 60%) and water content (5 and 10%). 1% of the drug was added to each formulation and examined.^6,12^

**Table 1:** Composition of selected ME formulation of finasteride

	**Factorial**	**S/C**	**% Oil**	**% S + C**	**Water**
ME-F-1	+ + +	3:1	60	30	10
ME-F-2	- + +	3:1	60	35	5
ME-F-3	+ - +	3:1	10	80	10
ME-F-4	- - +	3:1	10	85	5
ME-F-5	+ - -	2:1	10	80	10
ME-F-6	- - -	2:1	10	85	5
ME-F-7	- + -	2:1	60	35	5
ME-F-8	+ + -	2:1	60	30	10

### Polarized light microscopy

To corroborate the isotropic nature of MEs, the samples were examined using a polarized cross-sectional microscope (Olympus BX51 U-AN 360, Tokyo, Japan). A drop of ME sample was placed between the front and a glass slide to be observed under a polarized cross light. The isotropic material, such as a ME does not interfere with polarized light compared with anisotropic liquid crystals and a dark field of vision.^13,14^

### Preparation of finasteride microemulsions

**Figure 1:**
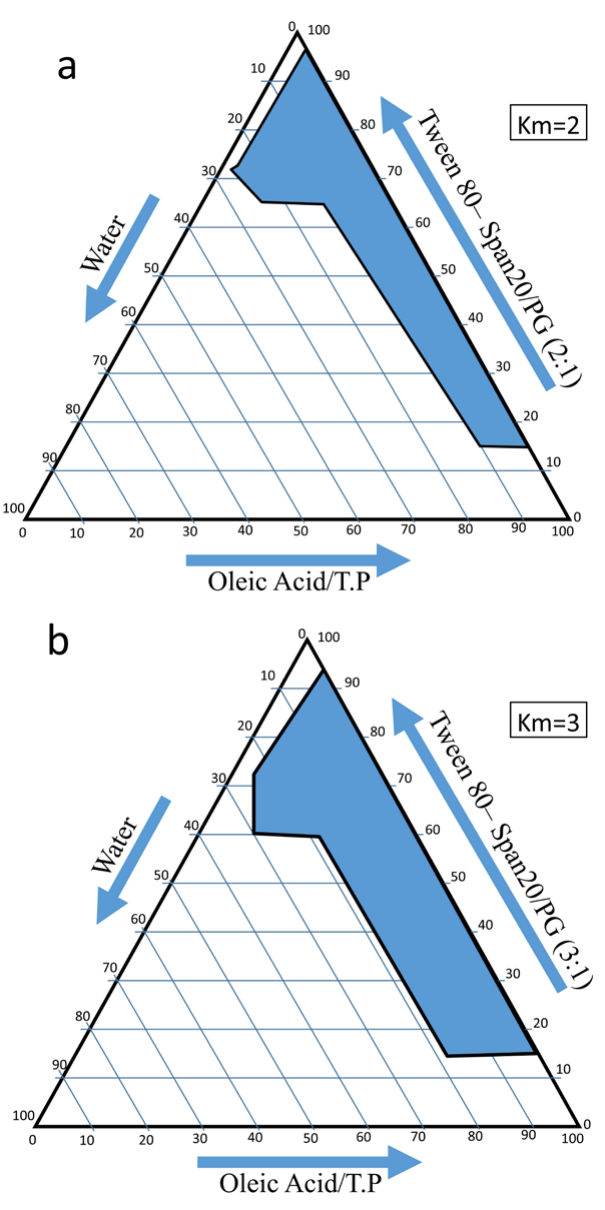
The pseudo ternary phase diagrams of the oil-surfactant/cosurfactant mixture-water system at the 2:1 and 3:1 weight ratio of Tween 80/Span 20/PG at ambient temperature, blue area show microemulsions zone.

MEs were employed for the construction of phase diagrams (Figure 1) and full-factor design with three variables at two levels. Independent variables including the ratio of surfactant to co-surfactant (s/c), oil proportion and water proportion. Factorial design of eight formulations with high and low levels of oil (60% and 10%), water (10% and 5%), surfactant to corn ratios (2:1 and 3:1) and finasteride (1%) were prepared. Further, finasteride was added to a mixture of oil, then in a mixture of surfactants and co-surfactants; enough drops of distilled water were added to the mixture, which was then further mixed with the finasteride containing ME.^15,16^

### Droplet size determination

The droplet size of each of the MEs was evaluated by a particle size analyzer (Scatter Scope 1 Quidex, South Korea). The mean droplet size and dispersion index were measured.^15,17^

### pH and viscosity measures

The samples were examined for viscosity. For this purpose, at 25 ± 0.5° C and with a Brookfield viscometer (Brookfield Engineering, Middleboro, Massachusetts, USA) , DV-II and Spindel 34 were measured in 10 ml volumes at shear rates of 75 rpm.18 The pH of the samples was measured with a Mettler (Mettler Toledo, Wan, Kowloon, Hong Kong) pH meter at 25° C without dilution.

### Differential scanning calorimetry (DSC)

A Mettler-Toledo DSC (Mettler Toledo, Wan, Kowloon, Hong Kong) was made use of to carry out the thermal analysis of MEs. For this purpose, a small amount of specimen was weighed in aluminum foam, and then the doors were completely closed so that they did not exchange any material with the outside environment. DSC was performed in cooling mode.15 In cooling mode, the samples were exposed to a temperature of +30° C to -50° C and a scan speed of 5°C/min. The rate of decrease in temperature was 5°C/min in this method.19 In this case, aluminum foam was used as a reference.

### Drug release study

Different formulations were utilized to investigate drug release from the Franz diffusion cells with a cross-sectional area of 3.4618 cm^2^. With this method, mixture buffer phosphate (pH = 7) and methanol (2:1) were selected as the receptor phase. An artificial cellulose membrane that was soaked in deionized water for 24 hours prior to testing was employed as a membrane model. To perform the test, the receiver was filled with 30 ml of receiver phase and placed on the styrene device at a temperature of 37 ± 0.5°C and with a magnet rate of 200 rpm. Next, 3 g of each formulation was weighed and spread on the membrane. The temperature setting of the receiver phase was set by the device.

At time zero, the magnet was turned on and contact with the membrane was initiated, being removed at specified intervals (0.5, 1, 2, 3, 4, 5, 6.7, 8 and 24 hours) from the receptacle; 2 mL of fresh solution was replaced by the receptor phase, and the amount of the drug was determined by a spectrophotometric method at 224 nm wavelength. The study was repeated three times for each of the examples.^20^

In order to establish the kinetic model of drug release, zero-order, first-order and Higuchi models were fit to the results, and the highest r^2^ was the criterion for selecting the model.

### Physical stability of MEs

With different formulations, several samples were prepared in 5 mL volumes and stored at 4, 25 and 37°C for six months. During this period, samples were examined each week for apparent characteristics (transparency, uniformity and droplet size variation). Any change, such as in the appearance of turbidity or separation of phases in formulations, was a sign of instability.^21-23^

### Permeability experiments

Until the permeability testing, the skin was kept in a freezer. Before use, the skin samples were removed from the freezer and kept at room temperature to reach room temperature. Thereafter, they were cut into small pieces and placed on diffusion cells such that the stratum corneum was placed into donor phase and hydrated at 37°C for 16 hours between the phases of the receptor and donor, then the phases were evacuated. Inside the receptor with a volume of 22 mL, the receiver phase was poured out and 5 g of finasteride ME was poured into the compartment and a finasteride passage test was conducted on the entirety of the rat skin. However, the thickness of the samples and amount of hydration of the skin before and after initial contact with the phase of the receptor were measured.

To investigate the effect of MEs, after hydrating and placing the skin on Franz cells, 5 g of ME formulations were placed on the skin, and the receptor phase was filled with phosphate buffer (pH =7) and methanol (1:2) and placed on styrene. The receiver phase was stirred at 200 rpm with a magnet. At specific times (0.5, 1, 2…, 8, 24, 26, 28, 32, 36 and 48), sampling of the receiver phase was performed and 2 ml of the receiver phase was removed immediately at equal volumes from the fresh solvent to replace the sink conditions. The amount of drug was measured by UV spectroscopy at 224 nm. An ME without drug was used as a control.^24^

### Data analysis and statistics

The analysis of variance (ANOVA) method was employed to analyze the difference between the parameters of the permeation of finasteride in ME formulations. Minitab 17 software (Minitab, Inc, Pennsylvania, USA) was utilized to evaluate the effects of independent variables on dependent variables. Using Sigma Plot version14 software (Systat Software, Inc, San Jose, California, USA), for the proposed ratios, ternary phase diagrams were plotted and the best proportions that yielded the ME formulation were determined.

All studies were repeated three times, and values were expressed as mean of standard deviation. For statistical analysis, two-way *t* tests and ANOVAs were applied. To design the full-factorial test, Minitab 17 was used.

Enhancement ratios (ER) were calculated from equation 1.

(1) $$ER=\frac{\text{Permeability parameter after treatment}}{\text{Permeability parameter before treatment}}$$

In this study, the penetration rate of finasteride in MEs was studied from whole rat skin and permeability parameters, such as passage rate in an equilibrium state (Jss), permeability coefficient (p), lag time (Tlag) and apparent diffusivity coefficient (Dapp), was calculated. In order to establish cross-flow parameters, cumulative volume of the drug passed through the surface unit versus time was plotted. The permeability coefficient (p) was calculated from equation 2^25^:

(2) $${\text{J}}_{ss}=\text{P}\times \text{C}$$

*C*: Drug concentration in the donor phase.

*Tlag*: clamping time of drug obtained from the skin along the line of equilibrium to the axis of time in the cumulative curve of the drug.

The value of *D* is calculated from Equation 3^26^:

(3) $$D=\frac{{\text{h}}^{2}}{6\text{Tlag}}$$

As *h* does not represent the actual length of the pathway, the *D* calculated from this formula is also apparent to D. Seeing all calculations are based on the steady-state region, the cumulative flow rate of the drug is determined, so the establishment of sink conditions is indispensable for the citation of these parameters. In this work, the maximum concentration established in the receptor phase was less than 10% of the saturation solubility of the drug in the receptor phase, and therefore, a steady concentration gradient was established during the experiments, and with these conditions, a steady passage rate was computed.

## Results

### Solubility of finasteride

The solubility of finasteride is found in Table 2.

**Table 2:** Results of drug solubility in each of the components of microemulsion formulation (mean ± SD, n = 3)

**Phase type**	**Excipient**	**Solubility (mg/mL)**
Oil	Oleic acid	8.1 ± 0.1
Oil	Transcutol P	15 ± 0.2
Oil (mixture)	Oleic acid+Transcutol P	20.1 ± 0.4
Surfactants	Span20	3.2 ± 0.1
Surfactant	Tween 80	2.9 ± 0.2
Co-surfactant	PG	11 ± 0.3

### Phase studies

**Figure 2:**
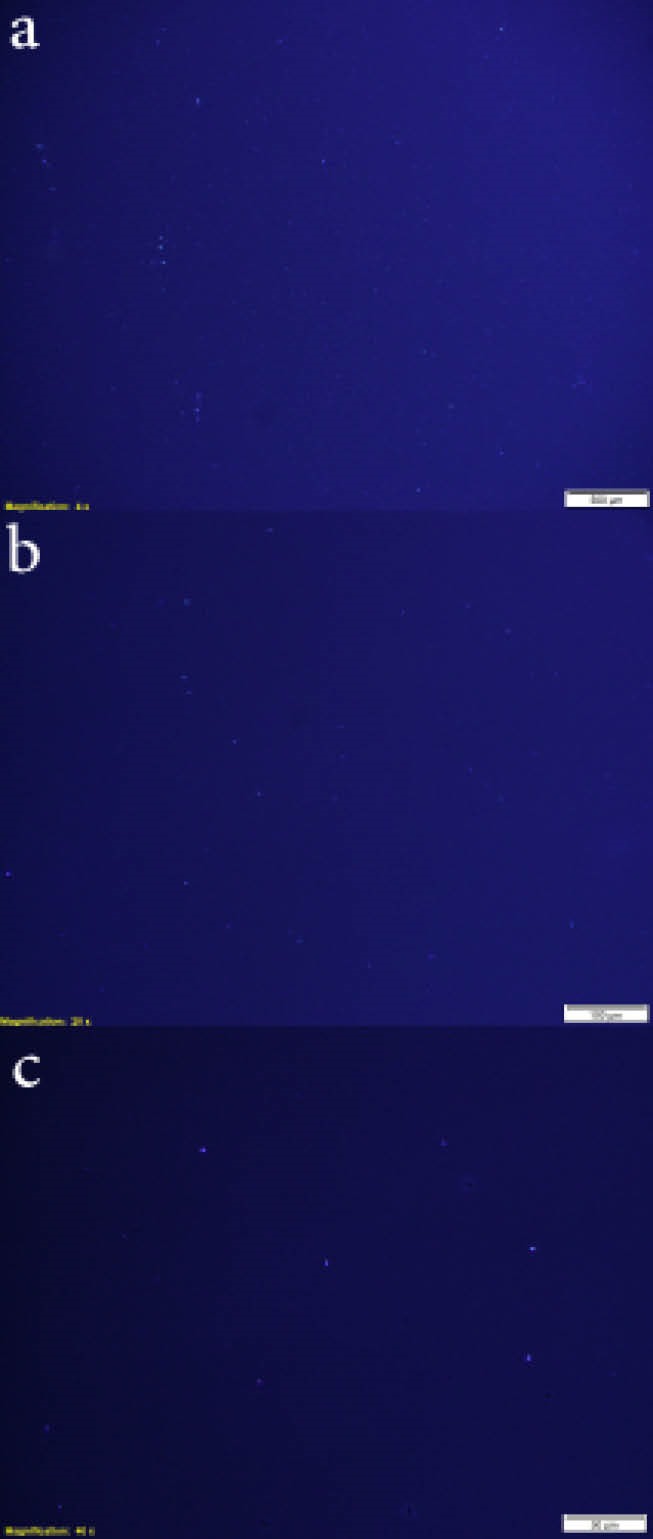
The image of the polarizing microscope from ME-F-4 in magnifications 4 (a), 20 (b) and 40 (c).

The pseudo-ternary phase diagrams of oleic acid-Transcutol P/Span 20-Tween 80/PG/water are presented in Figure 1. ME structures were observed using cross-polarized light microscopy. Figure 2 depicts the black background for all formulations of MEs under polarized light microscopy.

### Characterization of finasteride MEs

The viscosity, mean droplet size, polydispersity index (PI) and pH of finasterideMEs are located in Table 3.

**Table 3:** Physicochemical parameters of MEs (mean ± SD, n = 3)

	**pH**	**Viscosity (cps)**	**Mean droplet size (nm)**	**Polydispersity index**
ME-F-1	5.1 ± 0.1	97 ± 2.6	7.74 ± 1.6	0.328 ± 0.016
ME-F-2	5.2 ± 0.2	86.4 ± 3.4	7.12 ± 1.2	0.333 ± 0.022
ME-F-3	5.4 ± 0.1	209.6 ± 8.2	12.5 ± 1.4	0.354 ± 0.008
ME-F-4	5.4 ± 0.3	202.4 ± 3.8	16.95 ± 0.9	0.334 ± 0.016
ME-F-5	5.6 ± 0.3	193.6 ± 4.5	11.6 ± 0.1	0.321 ± 0.0015
ME-F-6	5.6 ± 0.1	183.3 ± 6.2	5.5 ± 2.4	0.333 ± 0.002
ME-F-7	5.7 ± 0.2	96 ± 3.4	13.14 ± 1.8	0.338 ± 0.002
ME-F-8	5.7 ± 0.1	104 ± 2.6	15.5 ± 5.4	0.339 ± 0.003

The amount and kinetics underlying drug release from the MEs are presented in Table 4.

**Table 4:** Percentage release and kinetic release of selected MEs (mean ± SD, n = 3)

	**Kinetic of release**	**r** ^ 2 ^	**Q**_2h(%)_	**Q**_24h(%)_
ME-F-1	Higuchi	0.9897	10.6 ± 5.4	39.34 ± 1.11
ME-F-2	Higuchi	0.9857	7.918 ± 2.185	32.201 ± 4.039
ME-F-3	first	0.9966	7.457 ± 3.572	48.158 ± 1.250
ME-F-4	first	0.9946	5.977 ± 0.346	46.861 ± 2.704
ME-F-5	first	0.9985	4.897 ± 0.798	48.724 ± 2.430
ME-F-6	first	0.9992	5.303 ± 0.438	49.510 ± 3.292
ME-F-7	Higuchi	0.9933	6.402 ± 0.883	28.668 ± 4.903
ME-F-8	first	0.9906	6.230 ± 1.078	31.134 ± 0.086

The results show that the percentage of 24-hour release of the drug from the MEs in formulation 6 is the highest and lowest in ME 7.

Thermal analysis was conducted on finasteride MEs by cooling. The results from determining the phase transition temperature and enthalpy of finasteride MEs in the cooling program are provided in Table 5.

**Table 5:** Transition temperature and enthalpy of ME formulations of finasteride (mean ± SD, n = 3)

	**T**_m2_ **(°C))Melting Point)**	**ΔH (mj/mg)**
ME-F-1	-27 ± 0.1	44.22 ± 1.1
ME-F-2	-27 ± 0.2	50.43 ± 0.9
ME-F-3	-30 ± 0.5	1.01 ± 0.11
ME-F-4	-31 ± 0.4	0.64 ± 0.12
ME-F-5	-31 ± 0.3	0.96 ± 0.15
ME-F-6	-32 ± 0.5	0.62 ± 0.1
ME-F-7	-48 ± 0.5	26.88 ± 1.1
ME-F-8	-33 ± 0.2	29.73 ± 1.3

It has been shown that all finasteride ME formulations have appropriate characteristics with regards to their homogeneity and stability over six months.

The permeability parameters of the finasteride ME formulations in comparison with saturation controls are given in Table 6.

**Table 6:** permeability parameters of finasteride microemulsions compared with control ( mean ± SD, n = 3)

	**J**_ss_ **(mg/cm2.h)**	**D**_app_ **(cm** ^ 2 ^ **/h)**	**P(cm/h)**	**T**_lag_ **(h)**	ER*_p_	**ER**_flux_	**ER**_D_	**ER**_p_
Control	0.0046 ± 0.0051	0.0116 ± 0.0006	0.00045 ± 0.0005	4.67 ± 0.241	-	-	-	-
ME-F-1	0.0322 ± 0.0126	0.0164 ± 0.0120	0.0032 ± 0.0012	4.54 ± 0.345	21.97 ± 2.9	22.02 ± 1.2	1.38 ± 0.9	21.97 ± 2.9
ME-F-2	0.0169 ± 0.0010	0.0100 ± 0.0075	0.0017 ± 0.0001	7.53 ± 0.665	9.18 ± 0.8	9.18 ± 0.9	0.88 ± 0.6	9.18 ± 0.8
ME-F-3	0.0107 ± 0.0009	0.0574 ± 0.0130	0.0011 ± 8×10^-5^	1 ± 0.2	6.3 ± 1.13	6.31 ± 0.19	4.98 ± 1.3	6.3 ± 1.13
ME-F-4	0.0194 ± 0.0009	0.0066 ± 0.0018	0.0019 ± 8×10^-5^	8.48 ± 1.2	11.14 ± 2.458	11.1 ± 2.5	0.5617 ± 0.12	11.14 ± 2.458
ME-F-5	0.0326 ± 0.0060	0.0202 ± 0.0026	0.0032 ± 0.0006	2.69 ± 0.355	16.3 ± 2.6	16.45 ± 1.9	1.74 ± 0.14	16.3 ± 2.6
ME-F-6	0.0456 ± 0.0024	0.8086 ± 0.12	0.0045 ± 0.0003	1.65 ± 0.290	26.35 ± 2.5	26.32 ± 2.6	67.25 ± 3.02	26.35 ± 2.5
ME-F-7	0.0241 ± 0.0066	0.0196 ± 0.005	0.0024 ± 0.0006	2.85 ± 0.7	15.59 ± 1.6	15.58 ± 1.6	1.704 ± 0.51	15.59 ± 1.6
ME-F-8	0.0281 ± 0.0205	0.0252 ± 0.0261	0.0028 ± 0.0020	4.6 ± 0.790	22.13 ± 2.9	22.12 ± 2.94	2.117 ± 0.140	22.13 ± 2.9

## Discussion

For introducing the appropriate formulation of finasteride ME, as well as introducing a mixture of oil, surfactants and appropriate co- surfactants, the degree of solubility of finasteride in each component was employed. Our study showed that mixtures of oleic acid and Transcutol P with a ratio of 10:1 as oil, mixtures of Span 20 and Tween 80 as surfactants and polyethylene glycol as co-surfactants were suitable. In addition, the phase diagram showed that with increasing the ratio of surfactants to co-surfactants, the width of the ME region increased.^27^

The droplet size of the MEs was in the range of 5.50 to 16.95 nm, which is within the range of MEs (1-100 nm).^28^ The relationship between the size of the droplet and independent variables has shown that the droplet size of MEs was not significant with any of the parameters.^29^ The particle size dispersion index (PDI) indicated the uniformity of droplet size in the MEs; The PDI values obtained in this study were less than 0.5 for all MEs. Therefore, the results indicated the uniformity of droplet size of the MEs. These results were consistent with the conclusions of previous articles.^13^

The viscosity of the MEs was within the range of 86.4 to 209.6 cps. Analysis of data variance showed that there is a significant relationship between the oil content (*P* < 0.05) and theconstant of the equation (*P* < 0.05), thus increasing the oil content leads to a rise in viscosity of the desired formulations. Additionally, given that equation *P* is less than 0.05, other formulation components also affected viscosity for the formulation of each ME.

The pH of the MEs was within the range of 5.1-5.7. Analysis of data variance suggested that the pH of the formulation with oil proportion (*P* = 0.170) and water content (*P* = 0.640) was not significant, but with the ratio of s/c (*P* < 0.05) and constant of the equation (*P* < 0.05), there was significance. Reduction of the s/c ratio increased pH. Moreover, owing to the significance of the constant of the equation, other formulation components were noted to influence pH. The studied MEs had suitable pH for the skin.

Drug release of finasteride MEs followed different kinetics. The analysis of the variance of the data for the MEs showed that the relationship between the percentage of drug release from the MEs with the oil content and ratio of s/c, the constant water content of the equation was not significant. The maximum amount of drug release in two hours happened in formulation ME-F-1, and the lowest amount of drug release at the second hour happened in formulation ME-F-5.

Diminishing oil content enhanced the release of the drug after 24 hours. ME-F-6 (containing 5% (w/w) water, 56.67% (w/w) surfactant, 10% (w/w) oil phase and 28.33% (w/w) co-surfactant) and ME-F-5 (containing 10% (w/w) water, 53.34% (w/w) surfactant, 10% (w/w) oil-phase and 26.66% (w/w) co-surfactant), which had a liberation proportion of 49.510 and 48.724%, respectively, the highest degree of release among the studied formulations. Based on the analysis of the variance of drug release data with the MEs, the percentage of drug release from MEs with oil content (*P* < 0.05) and constant of the equation (*P* < 0.05) were significant, while with other variables, there was no significance. Therefore, by increasing the proportion of oil in the MEs, the percentage of drug release increased after 24 hours. With high water levels, the amount of drug release was increased with a rising s/c ratio.

DSC studies were used in the cooling program to study the water behavior of the MEs. The water found in the MEs was both free water (bulk) and bonded water (between the surface). There are several reports of phase transfer temperatures for free water and water bonded in the DSC experiments.^30^

Podlogar et al reported limited free water at temperatures ranging from -8°C to 0°C and limited bonded water at temperatures ranging from -26° C to -17°C, demonstrating that if the water content is low in a ME, the freezing point of the bonded water was very low. The bottom (-45°C) showed that the water was tightly bonded to the surfactant. The distilled water employed had a sharp and large carrier in the range of -17°C, which was referred to as the freezing point of super frozen water. This water does not interact with other molecules in the ME system.^31^

The study of finasteride ME formulation thermograms showed that for all MEs, the free-flowing or bulk temperature range was at 0°C, but all thermograms had an exothermic peak in the range of -48°C to -27°C. This is related to water-bonded water, which is strongly bound to surfactant.30 The presence of the phase transition temperature in ME-F-1 in the range of -48° C indicated that the water was firmly attached to the surfactant, which is consistent with the results of Podlogar and colleagues.^31^

Bound melting transition temperature (Tm_2_) in finasteride MEs is related to the ratio of s/c (*P* < 0.05) such that with increasing s/c ratios, Tm_2_ increases. The constant of the equation (*P* < 0.05) with Tm_2_ shows that other materials in the formulation also affect this temperature.

Furthermore, with a high proportion of oil, increasing water content from 5% to 10% reduced the freezing point of water. On the other hand, a high percentage of oil rose with an increasing s/c ratio from 2 to 3 such that Tm_2_ increased.

Based on the analysis of the DSC cooling curves of finasteride MEs, it was found that there was no significant relationship between water content and entangled water (H2) enthalpy, but with the equation constant (*P* < 0.05), oil proportion (*P* < 0.05) and s/c ratio (*P* < 0.05), there was significance. Considering the ability of oleic acid as the oil phase in the ME studied in terms of hydrogen bonding, it seems that the bonding effect on the thermal behavior of water in MEs is likely to be influenced.

Yet, the percentage of oil proportion, water proportion and s/c ratio, free radicals experience open flux in MEs so that the increase in free enthalpy rose with an increase in either of the three parameters. Concerning the enthalpy of bonded water, oil proportion and water proportion, there were significant effects, so by increasing the percentage of oil and reducing water content, enthalpy of bonded water increased.

In order to investigate the stability of MEs, a narrow PDI value was observed for the formulations of finasteride. This parameter could indicate the degree of stability of the carrier. MEs were uniformly prepared with a clear dispersion and without separation of phases after centrifugation. In previous studies on the sustainability of MEs, it has been shown that there is a complex relationship between zero interfacial tension and thermodynamic stability.^32^ Thus, finasteride MEs could protect a drug from degradation for a long time without antioxidants.

ME vehicles have been able to influence certain formulations in terms of distribution. A number of these vehicles have been seen to increase the distribution of the drug by 67-fold over the saturation control.

Our results indicated that the effect of ME structure had a greater effect on the amount of flux and P, mainly because of the increase in the oil phase and amount of surfactant formulation, increasing the amount of flux and P in ME with a high probability most likely because of the fluidization of the lipid matrix or loss of structure of the lipid tissue of the horn tissue by Oleic acid and the surfactant formulation system.

Previous studies have shown that Oleic acid acts as an absorption enhancer in MEs, causing intermittent lipid disturbances, and eventually a separate lipid phase as a pond within the peripheral space, facilitating the passage of the drug.^33^ On the other hand, oleic acid is likely to dissolve the ligation of the horny layer by diminishing the lipid-binding temperature.^33,34^

Earlier work has clearly demonstrated the effect of adsorption on unsaturated fatty acids was greater than that of saturation, and among non-saturated fatty acids, dual bonding compounds exhibited a higher absorption effect. Cis compounds also have a more attractive influence on trans-space arrangement.^35^ Based on the presence of a non-saturated band with cis spatial arrangement because of the difference in makeup relative to the lipid chains present in the bilayer interlayer structure after entry, there is an induction of irregularities and reduction of the transition temperature of the gel phase to liquid crystal.^36^

Propylene glycol, with an effect similar to ethanol, accelerates the distribution of the drug in the stratum corneum and, to a lesser extent, disturbs the structure of cellular fats. The use of 10% propylene glycol, along with oleic acid, increases the effect of uptake.^36^ The absorption in different formulations varies.^35^ This agent has its own effects by locating keratin in the stratum corneum via occupation of hydrogen bonding sites.^37^

Surfactants have a direct impact on the properties of the skin barrier and indirect effects on the thermodynamic properties of drug substances from the vehicle to the skin. These thermodynamic properties lead to the release of the drug into the skin. Surfactant monomers penetrate into the skin by interfering with it, altering the properties of the skin barrier and facilitating the entry of the drug into the skin.^38^ Non-ionic surfactants change the power distribution of the drug in the skin.^39^ Surfactants enhance penetration of the drug by dissolving stratum corneum lipids.^40^ The strength of surfactant binding to proteins within the horn layer leads to an increase in their absorption properties.^41^ They also interfere with the keratin of the stratum corneum cells.^42^ Span 20 (sorbitan monolaurate) is a non-ionic surfactant that has strong absorption in the skin.^35^

The effect of MEs on the transfer of drug from rat skin compared to control (1% suspension) was calculated by calculating ER_Flux_, ER_P_ and ER_D_. The results showed that all ME formulations caused a significant increase in ERFlux, ER_P_ and ER_D._

The relationship between the permeability parameters of the skin and independent variables showed that the parameters of T_Lag_ and J_ss_ with independent variables in the studied range were not significant.

In addition, the results showed that the P coefficient had a significant relationship with the constant of the equation, and with a modification to other components, the value of P can be changed.

The relationship between independent variables and apparent diffusivity coefficient (D_app_) shows that this parameter (D_app_) has a significant relationship with water content, so that with increasing water content, D_app_ increases.

The results have shown that all ME formulations have a greater effect on flux than the difiusivity coefficient.

According to the results presented herein, it was found that ME-F-6 had the highest rate of drug passage (J_ss_) was 26.32 times more than the control group, therefore being the most suitable formulation for transfusion from rat skin.

## Conclusion

The current study showed that any change in the content and composition of MEs could change physicochemical characteristics and drug permeability parameters.

The selection of ME vehicle to a large extent may increase the stability of finasteride and help pass the drug through the skin. The kinetics of drug release from the vehicle were based on the Higuchi or first-order models, indicating a long release versus finasteride solution.

## Ethical Issues

Not applicable.

## Conflict of Interest

The publication has been approved by all co‐authors and the responsible authorities at the institute where the work has been carried out.

## Acknowledgments

This article was extracted from the pharm.D thesis (Mohammad Soleymani) and was sponsored by Ahwaz University of Medical Sciences, Jundishapur, Ahwaz.

## References

[1] Sakr FM, Gado AMI, Mohammed HR, Adam ANI (2013). Preparation and evaluation of a multimodal minoxidil microemulsion versus minoxidil. Drug Des Devel Ther.

[2] Danielsson I, Lindman B (1981). The definition of microemulsion. Colloids Surf.

[3] Karrer-Voegeli S, Rey F, Reymond MJ, Meuwly JY, Gaillard RC, Gomez F (2009). Androgen dependence of hirsutism, acne, and alopecia in women: Retrospective analysis of 228 patients investigated for hyperandrogenism. Medicine (Baltimore).

[4] Lindman B, Stilbs P, Moseley ME (1981). Fourier transform nmr self-diffusion and microemulsion structure. J Colloid Interface Sci.

[5] Boonme P, Kaewbanjong J, Sangduangyang S, Suksawat S, Teeranachaideekul V (2016). Microemulsion-based hydrogels of itraconazole: Evaluation of characteristics and stability. Asian J Pharm Sci.

[6] Du Z, Mao X, Tai X, Wang G, Liu X (2016). Preparation and properties of microemulsion detergent with linear medium chain fatty alcohols as oil phase. J Mol Liq.

[7] Salimi A, Hedayatipour N, Moghimipour E (2016). The effect of various vehicles on the naproxen permeability through rat skin: A mechanistic study by DSC and FT-IR techniques. Adv Pharm Bull.

[8] Moghimipour E, Salimi A, Sharif Makhmal Zadeh (2013). Effect of the various solvents on the in vitro permeability of vitamin B12 through excised rat skin. Trop J Pharm Res.

[9] Israelachvili JN, Mitchell DJ, Ninham BW (1976). Theory of self-assembly of hydrocarbon amphiphiles into micelles and bilayers. Journal of the Chemical Society Faraday Transactions 2.

[10] Acharya SP, Pundarikakshudu K, Panchal A, Lalwani A (2013). Preparation and evaluation of transnasal microemulsion of carbamazepine. Asian J Pharm Sci.

[11] Magdassi S, Ben Moshe M, Talmon Y, Danino D (2003). Microemulsions based on anionic gemini surfactant. Colloids Surf A Physicochem Eng Asp.

[12] Salimi A, Moghimipour E, Tavakolbekhoda N (2013). Transdermal delivery of celecoxib through rat skin from various microemulsions. Int Res J Pharm App Sci.

[13] Moghimipour E, Salimi A, Changizi S (2017). Preparation and microstructural characterization of griseofulvin microemulsions using different experimental methods: SAXS and DSC. Adv Pharm Bull.

[14] Djekic L, Primorac M, Jockovic J (2011). Phase behaviour, microstructure and ibuprofen solubilization capacity of pseudo-ternary nonionic microemulsions. J Mol Liq.

[15] Gee G, Bauder J, Klute A. Methods of Soil Analysis: Part 1—Physical and Mineralogical Methods. Madison, WI: Soil Science Society of America, American Society of Agronomy; 1986.

[16] Poomanee W, Chaiyana W, Randall Wickett R, Leelapornpisid P (2017). Poomanee W, Chaiyana W, Randall Wickett R, Leelapornpisid PStability and solubility improvement of sompoi (Acacia concinna Linn) pod extract by topical microemulsion. Asian J Pharm Sci.

[17] Hargreaves AL, Gregson F, Kirby AK, Engelskirchen S, Bain CD (2015). Microemulsion droplets in optical traps. J Mol Liq.

[18] Crooks GE, Rees GD, Robinson BH, Svensson M, Stephenson GR (1995). Crooks GE, Rees GD, Robinson BH, Svensson M, Stephenson GRComparison of hydrolysis and esterification behavior of Humicola lanuginosa and Rhizomucor miehei lipases in AOT-stabilized water-in-oil microemulsions: IiEffect of temperature on reaction kinetics and general considerations of stability and productivity. Biotechnol Bioeng.

[19] Garti N, Aserin A, Ezrahi S, Tiunova I, Berkovic G (1996). Water behavior in nonionic surfactant systems I: Subzero temperature behavior of water in nonionic microemulsions studied by DSC. J Colloid Interface Sci.

[20] Eastoe J, Paul A, Downer A, Steytler DC, Rumsey E (2002). Eastoe J, Paul A, Downer A, Steytler DC, Rumsey EEffects of fluorocarbon surfactant chain structure on stability of water-in-carbon dioxide microemulsionsLinks between aqueous surface tension and microemulsion stability. Langmuir.

[21] Zarghi A, Jenabi M, Ebrahimian AJ (1998). HPLC determination of the stability of tretinoin in tretinoin–minoxidil solution. Pharm Acta Helv.

[22] Cieśla J, Koczańska M, Narkiewicz-Michałek J, Szymula M, Bieganowski A (2017). Effect of α-tocopherol on the properties of microemulsions stabilized by the ionic surfactants. J Mol Liq.

[23] Kartsev VN, Shtykov SN, Bogomolova IV, Ryzhov IP (2009). Thermodynamic stability of microemulsion based on sodium dodecyl sulfate. J Mol Liq.

[24] Megrab NA, Williams AC, Barry BW (1995). Oestradiol permeation through human skin and silastic membrane: Effects of propylene glycol and supersaturation. Journal of Controlled Release.

[25] Spicer PT, Small WB, Small WB, Lynch ML, Burns JL (2002). Dry powder precursors of cubic liquid crystalline nanoparticles (cubosomes). J Nanopart Res.

[26] Krauel K, Girvan L, Hook S, Rades T (2007). Characterisation of colloidal drug delivery systems from the naked eye to Cryo-FESEM. Micron.

[27] Rahdar A, Almasi-Kashi M, Khan AM, Aliahmad M, Salimi A, Guettari M (2018). Effect of ion exchange in NaAOT surfactant on droplet size and location of dye within Rhodamine B (RhB)-containing microemulsion at low dye concentration. J Mol Liq.

[28] Slomkowski S, Alemán José V, Gilbert Robert G, Hess M, Horie K, Jones Richard G (2011). Terminology of polymers and polymerization processes in dispersed systems (IUPAC recommendations 2011). Pure and Applied Chemistry.

[29] Mehnert W, Mader K (2001). Solid lipid nanoparticles: Production, characterization and applications. Adv Drug Deliv Rev.

[30] Garti N, Aserin A, Tiunova I, Fanun M A DSC study of water behavior in wate- in – oil microemulsions stabilized by sucrose esters and butanol. Colloids Surf A Physicochem Eng Asp.

[31] Podlogar F, Gasperlin M, Tomsic M, Jamnik A, Rogac MB (2004). Structural characterisation of water-Tween 40/Imwitor 308-isopropyl myristate microemulsions using different experimental methods. Int J Pharm.

[32] Naveh N, Weissman C, Muchtar S, Benita S, Mechoulam R (2000). A submicron emulsion of hu-211, a synthetic cannabinoid, reduces intraocular pressure in rabbits. Graefes Arch Clin Exp Ophthalmol.

[33] Ongpipattanakul B, Burnette RR, Potts RO, Francoeur ML (1991). Evidence that oleic acid exists in a separate phase within stratum corneum lipids. Pharm Res.

[34] Carpentieri-Rodrigues LN, Zanluchi JM, Grebogi IH (2007). Percutaneous absorption enhancers: Mechanisms and potential. Braz Arch Biol Technol.

[35] Trommer H, Neubert RH (2006). Trommer H, Neubert RHOvercoming the stratum corneum: The modulation of skin penetrationA review. Skin Pharmacol Physiol.

[36] Rolland A, Brzokewicz A, Shroot B, Jamoulle J-C (1991). Rolland A, Brzokewicz A, Shroot B, Jamoulle J-CEffect of penetration enhancers on the phase transition of multilamellar liposomes of dipalmitoylphosphatidylcholineA study by differential scanning calorimetry. Int J Pharm.

[37] Sinha VR, Kaur MP (2000). Permeation enhancers for transdermal drug delivery. Drug Dev Ind Pharm.

[38] Sarunyoo S (2009). An overview of skin penetration enhancers: Penetration enhancing activity, skin irritation potential and mechanism of action. Songklanakarin Journal of Science and Technology.

[39] Shen WW, Danti AG, Bruscato FN (1976). Effect of nonionic surfactants on percutaneous absorption of salicylic acid and sodium salicylate in the presence of dimethyl sulfoxide. J Pharm Sci.

[40] Cazares-Delgadillo J, Naik A, Kalia YN, Quintanar-Guerrero D, Ganem-Quintanar A (2005). Skin permeation enhancement by sucrose esters: A pH-dependent phenomenon. Int J Pharm.

[41] Breuer MM (1979). The interaction between surfactants and keratinous tissues. J Soc Cosmet Chem.

[42] Imokawa G, Akasaki S, Minematsu Y, Kawai M (1989). Importance of intercellular lipids in water-retention properties of the stratum corneum: Induction and recovery study of surfactant dry skin. Arch Dermatol Res.

